# Parathyroid Carcinoma in the Setting of Tertiary Hyperparathyroidism: Case Report and Review of the Literature

**DOI:** 10.1155/2020/5710468

**Published:** 2020-12-02

**Authors:** Federico Cappellacci, Fabio Medas, Gian Luigi Canu, Maria Letizia Lai, Giovanni Conzo, Enrico Erdas, Pietro Giorgio Calò

**Affiliations:** ^1^Department of Surgical Sciences, University of Cagliari, “Policlinico Universitario Duilio Casula”, 09042 Monserrato, Italy; ^2^Department of Cytomorphology, University of Cagliari, Cagliari, Italy; ^3^Division of Medical Oncology, Department of Internal and Experimental Medicine “F. Magrassi”, School of Medicine, University of Campania “Luigi Vanvitelli”, Cagliari, Italy

## Abstract

**Introduction:**

Parathyroid carcinoma is one of the rarest cancers in normal population, and it is extremely uncommon in the setting of tertiary hyperparathyroidism. Indeed, only 24 cases have been reported in the literature. *Presentation of the Case*. We report the case of parathyroid carcinoma in a 51-year-old man, with a history of end-stage renal disease due to a horseshoe kidney treated with haemodialysis since 2013. He came to our attention due to an increase in calcium and parathyroid hormone serum levels. Neck ultrasound (US) showed a solid hypodense mass, probably the right inferior parathyroid gland, with an estimated size of 25 × 15 × 13 mm; the 99mTc-sestamibi SPECT/CT scan revealed a large radiotracer activity area in the right cervical region, compatible with a hyperfunctioning right inferior parathyroid gland. So, a tertiary hyperparathyroidism diagnosis was made. In April 2018, resection of three parathyroid glands was performed. Histopathological examination demonstrated the right inferior parathyroid gland specimen to be a parathyroid carcinoma, due to the presence of multiple, full-thickness, capsular infiltration foci, and a venous vascular invasion focus. *Discussion*. Diagnosis of parathyroid carcinoma in tertiary hyperparathyroidism is remarkably complex because of the lack of clinical diagnostic criteria and, in many cases, is made postoperatively at histopathological examination.

**Conclusion:**

To date, radical surgery represents the mainstay of treatment, with a five- and ten-year survival rates overall acceptable.

## 1. Introduction

Tertiary hyperparathyroidism (THP) is, most commonly, the outcome of longstanding secondary hyperparathyroidism (SHP) due to chronic kidney disease and is defined as the autonomous and excessive secretion of parathyroid hormone (PTH) by the parathyroid glands, leading to persistent hypercalcemia and elevated PTH serum level, which values do not decrease even when the underlying renal disease is resolved, usually after a renal allograft [1].

SHP is generally caused by diffuse parathyroid hyperplasia, while the onset of THP is usually due to an autonomous PTH-producing adenoma and very rarely to a carcinoma [2].

Indeed, parathyroid carcinoma (PC) is one of the rarest cancers, with a prevalence of 0.005% of all malignancies in the USA [3].

PC is usually associated with primary hyperparathyroidism (PHPT), while it is extremely rare in the setting of secondary or tertiary hyperparathyroidism (THP) [4].

To the best of our knowledge, only 24 cases of THP caused by a PC have been described in the literature [4–10]; herein, we report the case of the 25^th^ patient affected by this disease.

## 2. Case Report

A 51-year-old man, with a history of 15 years haemodialysis due to a horseshoe kidney, was referred to our surgical unit in April 2018 because of a progressive increase of calcium and parathyroid hormone serum levels.

His past medical history included a right inguinal hernia repair in 1979 and congenital biliary dysplasia.

His medications included sevelamer 800 mg, one pill at breakfast time and two pills at lunch and dinner time; pantoprazole 15 mg daily; bicarbonate 500 mg daily; and polystyrene sulfonate (Kayexalate) one tablespoon every two days. According to our nephrologist, no further therapy with calcimimetic drugs or calcitriol was administered.

The calcium level at the time of our first evaluation was 10.7 mg/dl, while the PTH level was more than 2000 pg/ml. Other biochemical data are summarized in Table 1.

There were no clinical features of hyperparathyroidism, and no parathyroid crisis occurs at the time of diagnosis. Physical examination showed no pathological alteration of the neck.

Preoperative 99mTc-sestamibi SPECT/CT (MIBI scan) revealed a large radiotracer activity area behind the inferior right pole of the thyroid gland, compatible with a hyperfunctioning right inferior parathyroid gland.

Neck ultrasound (US) showed, in the right inferior parathyroid area, a solid hypodense mass with an estimated size of 25 × 15 × 13 mm, compatible with a large parathyroid adenoma, without suspicion of local invasion.

Considering the presence of hypercalcemia and elevated PTH serum level, with US and SPECT/CT evidence of pathological gland, a preoperative diagnosis of tertiary hyperparathyroidism was made.

At surgery, preincision intact PTH, measured with the Future Diagnostics STAT-IntraOperative-Intact-PTH immunoassay kit, was 2582 pg/ml. After the mobilization of the right inferior thyroid lobe, an enlarged right inferior parathyroid was found and surgically removed. Near the right inferior laryngeal nerve, another enlarged mass was discovered and removed. On the left side, a soft mass, presumed to be the left inferior parathyroid gland, was identified and surgically removed.

Twenty minutes after the right inferior parathyroid resection, intact PTH levels, intraoperatively measured, dropped to 334 pg/ml.

At final neck exploration, no other pathological parathyroid glands were found, and there were no signs of local invasion of the surrounding tissues for any of the excised samples.

**Figure 1:**
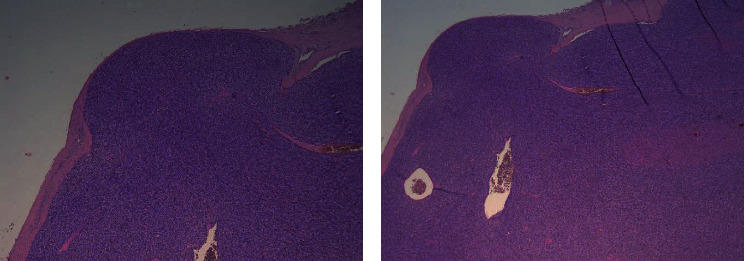
Section of the right inferior parathyroid specimen demonstrating a full-thickness capsular invasion (H&E, (a) 4x; (b) 2, 5x).

**Figure 2:**
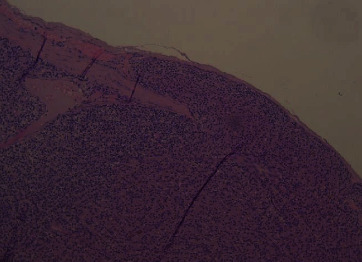
Section of the right inferior parathyroid specimen demonstrating a venous vascular invasion focus (H&E, 10x).

**Figure 3:**
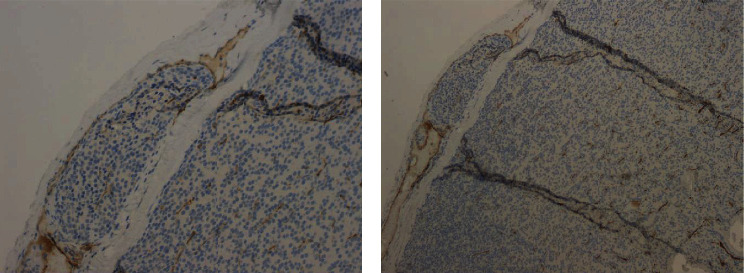
Section of the right inferior parathyroid specimen showing a venous vascular invasion focus highlighted by immunohistochemical staining for CD31 ((a) 20x; (b) 10x).

Histological examination revealed a parathyroid carcinoma on a 2.5 × 2 × 1.3 cm mass confirmed to be the right inferior parathyroid gland specimen. The tumour was circumscribed by a thick fibrous capsule, showing multiple full-thickness infiltration foci (Figure 1) and a venous vascular invasion focus (Figure 2), highlighted by immunohistochemical staining for CD31 (Figure 3). Furthermore, the lesion displayed frequent mitotic figures, with a positive Ki67 index in about 7-8% of the neoplastic element, but no fibrosis or necrosis was found. The pathology report confirmed all the other resected tissues to be diffuse parathyroid hyperplasia.

During follow-up, the patient had no postoperative complication.

Calcium and PTH serum levels on the first postoperative day were, respectively, 6.0 mg/dl and 101.4 pg/ml, while three days after the operation, they were 5.4 mg/dl and 283.9 pg/ml, respectively (Table 1).

These values are probably to be attributed to a hungry bone syndrome; therefore, calcium carbonate 2000 mg daily therapy was administered. The patient was finally discharged 3 days after the operation in good condition.

Currently, at 22 months of follow-up, which was performed by our nephrologists and endocrinologists and consisted of regular measurements of calcium and parathyroid hormone values, as well as further instrumental tests, aimed at investigating the possible presence of locoregional recurrence or distant metastases, he is free of disease.

## 3. Discussion

PC is a rare malignancy and generally presents an indolent but progressive behaviour [11].

According to the USA National Cancer Institute database, in nonrenal patients with PC, the age range at presentation was 15 to 55 years with a mean of 44 years [3]. At the time of diagnosis, the age of the 25 patients with parathyroid carcinoma ranged from 27 to 75 years, with a mean of 50, 4 years [4–10]; our patient received diagnosis when he was 51 years old.

Differently from PC in the setting of primary hyperparathyroidism, women and men are not equally affected: in fact, there is a clear female dominance (16 women and 9 men), but the reason remains unclear [4–12].

It is interesting to note that, as suggested by some authors, considering that almost 3% of PC has been diagnosed in haemodialysis patients, it seems to be more common in end-stage renal disease treated by haemodialysis than expected by chance [6, 13].

PC can be sporadic or in the context of genetic endocrine syndromes, such as hyperparathyroidism-jaw tumour syndrome (HPT-JT), multiple endocrine neoplasia type 1 (MEN 1), multiple endocrine neoplasia type 2A (MEN 2A), and familial isolated hyperparathyroidism (FIHP) [11, 12, 14, 15].

The aetiology of this tumour is unknown, but a genetic background has been established. Genomic alterations identified in PC are mostly represented by CDC73 gene mutations, the same gene involved in HPT-JT, codifying for a protein called parafibromin, which is liable, according to Cetani et al., for up to 70% of sporadic PC [11, 12, 14, 15]. Furthermore, evaluation regarding loss of parafibromin immunohistochemistry could be useful for diagnosis and differentiation of PC from other parathyroid lesions in daily practice, especially in cases with an initial suspicion of malignant potential to avoid excessive numbers of false positives [16–18]. In our patient, given the morphological alterations and other immunohistochemical investigation, our pathologists did not consider to perform parafibromin immunohistochemistry. Furthermore, assuming that the tumour was sporadic, neither the sequencing of CDC73 gene was carry out.

The diagnosis of PC is remarkably complex because of the lack of clinical diagnostic criteria and, in many cases, is made postoperatively at histopathological examination [12].

PC in tertiary hyperparathyroidism patients usually shows up with symptoms and signs of hyperparathyroidism, such as myalgia and arthralgia, weight loss, nephrolithiasis, and with bone disease localized in the spine, long bones, hands, and the skull [4–10].

As some authors suggest, in a patient with PHPT, severe hypercalcemia (>14 mg/dL), very high serum PTH levels (five times above the normal range), concomitant severe renal and skeletal manifestations, and palpable cervical mass should raise suspicion for PC [11, 12]. Nevertheless, calcium and PTH serum levels are not a useful indicator for the presence of PC in patients with THP, due to the end-stage renal disease: in fact, the kidney does not respond to parathyroid hormone in such cases [7].

Imaging techniques, such as neck ultrasound and ^99m^Tc-sestamibi scintigraphy, are useful for tumour localization but cannot reliably discriminate benign from malignant hyperparathyroidism [11, 12].

In case of preoperative suspicion for malignant disease, some authors recommend that a CT or MR imaging is performed [19]. It has also been suggested that other nuclear medicine techniques, such as 18F-Choline-PET, could be useful in the preoperative management of PC [20].

The finding of metastasis is the only certain criterion of malignancy [12].

Notably, among all cases reported in the literature, only one had preoperative PC suspect: Curto et al. had reported in 2019 a PC with a single pulmonary metastasis in a IV-stage chronic kidney disease patient, which was identified preoperatively with a combination of MIBI scan and PET-CT [10].

To our knowledge, this is the only preoperative diagnosis of PC in tertiary hyperparathyroidism, and in all the other cases, only intraoperative and/or histological findings made it possible to diagnose PC [4–10].

However, it is important to emphasize that also histopathological diagnosis may be difficult. Many pathological characteristics, such as trabecular architecture and mitotic figures, are not exclusive of PC, being also present in benign parathyroid diseases, while other histological features, such as capsular and vascular invasion or necrosis, are not always present in PC. The main challenge at histopathological examination is to distinguish PC from atypical adenoma. The latter tumour shares some histological characteristics with PC, such as fibrous septa, high mitotic activity, and diffuse growth pattern, but lack unequivocal signs of malignancy, as vascular, capsular, and/or perineural invasion [11, 12, 21]. In SHP, histological evaluation could be even challenging, due to the fact that the parathyroid glands tend to become larger, to develop multinodularity, and because of the greater presence of fibrous bands, which can complicate the evaluation of capsular invasion. So, in this setting, it is even more important to investigate the presence of vascular and/or perineural invasion before a PC diagnosis was given, to avoid the risk of false positive results.

As Erickson and Mete suggest, a panel of immunohistochemical markers may help in the histological evaluation of this tumour [22].

In our patient, the pathologist was able to diagnose PC thanks to the presence of multiple full-thickness capsular infiltration foci (Figure 1) and a venous vascular invasion focus (Figure 2), highlighted by immunohistochemical staining for CD31 (Figure 3), but, as we reported earlier, no fibrosis or necrosis was found.

Surgery is the mainstay of treatment, while usefulness of chemotherapy, radiotherapy, and other treatments remains still controversial. The surgical approach mostly suggested consists in an en-bloc resection of the pathological parathyroid gland with the ipsilateral thyroid lobe and any adjacent involved structures. Cervical lymph node dissection is mostly recommended in case of preoperative diagnosis or intraoperative suspicion of metastases [11, 12, 21].

Among the previous 24 patients with parathyroid cancer in the setting of tertiary hyperparathyroidism, 11 underwent parathyroidectomy alone, 5 underwent parathyroidectomy and neck lymphadenectomy, 5 underwent parathyroidectomy with total thyroidectomy, 2 underwent parathyroidectomy, lymphadenectomy, radical neck dissection, and wedge resection of some lung nodules, and 1 underwent parathyroidectomy and thyroid lobectomy [4–10].

In case of parathyroidectomy alone and histopathological diagnosis of PC, the management of the patient becomes more difficult. In this situation, clinical judgment and prompt decision making to choose further neck exploration or surveillance are required. In presence of aggressive histopathological features, such as extensive vascular and capsular invasion, a re-exploration to perform a more radical treatment should be indicated [11, 12, 23].

Restoration of normocalcemia after surgery indicates that all hyperfunctioning tissue has been removed. The use of intraoperative PTH determination could be helpful in surgical management [24–30].

Differently than PHPT, where requirements of success include normalization of PTH in addition to serum calcium levels, in THP the operative endpoint of surgery is not a return of PTH to normal value, but a drop in PTH level higher than 50% is considered sufficient, even if it remains above the normal range. Furthermore, the main objective of surgery for THP is not a normal PTH value, but a normal calcium level at least six months of follow-up [1].

As we reported in our patient, postoperative hypocalcaemia may occur, due to inhibition of remnant parathyroid glands or hungry bone syndrome, and, in this case, hypocalcaemia could be increased because of the chronic renal failure. This condition must be promptly treated with oral or intravenous calcium supplementation [11, 12].

PC recurrence occurs in more than 50% of patients, usually after 2-3 years from surgery, although relapses have been reported up to 23 years after initial treatment. For this reason, in patients with this tumour, follow-up should be careful and life-long [11, 12].

The recurrence site is most frequently locoregional, due to incomplete resection at initial surgery and cervical lymph node metastases; however, also distant metastases are not infrequent. Lymph node metastases occur in about 15–30% of patients at initial presentation. Distant metastases occur in about 25% of patients during follow-up, usually in the lungs, liver, and bone [11, 12]. Seven cases of disease recurrence on the total of 25 PC in the setting of THP are reported: the literature highlights five cases of lung metastasis, one case of liver metastasis, and one case of unknown recurrence origin [4–10].

Primary treatment for disease recurrence is surgical removal, both for local recurrences and, if possible, for distant metastases, even through repeated resection [11, 12, 23].

Five- and ten-year overall survival rates between 77–100 and 49–91%, respectively, have been described. Mortality generally is related to complications of hypercalcemia rather than from tumour burden [11, 12, 21].

In the renal dialysis cases, due to the small number of cases, it is difficult to establish a clear survival rate; however, the longest recorded survival is 9.5 years [31].

Regarding our patient, at 22 months of follow-up, he is alive and free of disease.

In conclusion, parathyroid carcinoma is an extremely rare tumour in the setting of THP, and his clinical diagnosis could be difficult due to the presence of chronic kidney failure, which leads to an increase in PTH blood level. Presence of metastasis is the only certain criterion of malignancies. To date, radical surgery remains the mainstay of treatment.

The recurrence rate of PC is generally high but is notable that 5- and 10-year survival rates are overall acceptable.

Notably in our patient, considering that the definitive diagnosis took place only postoperatively and given the absence of preoperative and intraoperative suspicion for local invasion, radical surgery was not performed.

## Figures and Tables

**Table 1:** Biochemical data at the time of diagnosis and after surgery.

	BS	1-day AS	2-day AS	3-day AS
Ca (mg/dl)	10.7	6.0	5.9	5.4
P (mg/dl)	6.8	4.3	4.2	4.4
iPTH (pg/ml)	>2000	101.4	226.9	283.9
25OH D (ng/ml)	15.2	—	—	—
Mg (mmol/l)	0.82	—	—	—
K (mEq/l)	5.3	4.6	—	—
Cr (mg/dl)	7.1	6.44	—	—
BUN (mg/dl)	65	52	—	—
ALP (U/l)	604	—	—	—
TSP (g/dl)	7.5	7.4	—	—

BS = before surgery; AS = after surgery. Ca = calcium; P = phosphorus; iPTH = intact parathyroid hormone; Mg = magnesium; K = potassium; Cr = creatinine; BUN = blood urea nitrogen; ALP = alkaline phosphatase; TSP = total serum protein.

## Data Availability

The data used to support the findings of this study are available from the corresponding author upon request.
